# HIV-1 Diversity and Drug Resistance Mutations among People Seeking HIV Diagnosis in Voluntary Counseling and Testing Sites in Rio de Janeiro, Brazil

**DOI:** 10.1371/journal.pone.0087622

**Published:** 2014-01-30

**Authors:** Carlos A. Velasco-de-Castro, Beatriz Grinsztejn, Valdiléa G. Veloso, Francisco I. Bastos, José H. Pilotto, Nilo Fernandes, Mariza G. Morgado

**Affiliations:** 1 Laboratório de AIDS & Imunologia Molecular, Instituto Oswaldo Cruz, Fundação Oswaldo Cruz, Rio de Janeiro, RJ, Brasil; 2 Instituto de Pesquisa Clínica Evandro Chagas, Fundação Oswaldo Cruz, Rio de Janeiro, RJ, Brasil; 3 Laboratório de Virologia, Departamento de Patologia Clínica, Instituto Fernandes Figueira, Fundação Oswaldo Cruz, Rio de Janeiro, RJ, Brasil; 4 Instituto de Comunicação e Informação Científica e Tecnológica em Saúde, Fundação Oswaldo Cruz, Rio de Janeiro, RJ, Brasil; 5 Laboratório de Informação em Saúde, Fundação Oswaldo Cruz, Rio de Janeiro, Brasil, and Imperial College London (CAPES visiting researcher # 17551-12-8), London, United Kingdom; 6 Hospital Geral de Nova Iguaçu, Nova Iguaçu, RJ, Brasil; Burnet Institute, Australia

## Abstract

The remarkable viral diversity remains a big challenge to the development of HIV vaccines and optimal therapy worldwide. In the latest years, as a consequence of the large expansion of highly active antiretroviral therapy (HAART) availability worldwide, an increase in transmitted drug resistance mutations (TDRM) has been observed, varying according the region. This study assessed HIV-1 diversity and TDRM profile over time among newly HIV-1 diagnosed individuals from Rio de Janeiro, Brazil. Blood samples were collected from individuals seeking HIV diagnosis in four voluntary counseling and testing (VCTs) sites located in the Rio de Janeiro Metropolitan Area, in 2005–2007. Recent (RS) and long-term (LTS) HIV-1 seroconverters were distinguished using BED-CEIA. Pol viral sequences were obtained for 102 LTS identified in 2005 and 144 RS from 2005–2007. HIV-1 subtype and *pol* recombinant genomes were determined using Rega HIV-1 Subtyping Tool and by phylogenetic inferences and bootscanning analyses. Surveillance of HIV-1 TDRM to protease and reverse transcriptase inhibitors were performed according to the Calibrated Population Resistance (CPR) Tool 6.0. Overall, subtype B remains the most prevalent in Rio de Janeiro in both LTS and RS HIV-1 infected individuals. An increased proportion of recombinant samples was detected over time, especially in RS heterosexual men, due to the emergence of CRF02_AG and URF samples bearing a subtype K fragment. The prevalence of HIV-1 samples carrying TDRM was high and similar between LTS and RS (15.7% *vs* 14.6%) or age (<25yo 17.9% *vs* >25yo 16.6%) along the study period. The high resistance levels detected in both populations are of concern, especially considering the dynamics of HIV-1 diversity over time. Our results suggest that the incorporation of resistance testing prior to HAART initiation should be highly considered, as well as permanent surveillance, aiming to carefully monitoring HIV-1 diversity, with focus on CRF/URF emergence and putative transmission.

## Introduction

HIV remains a global health problem, with an estimated 34.0 million people living with the virus in 2011 [1]. Although the global prevalence rate has remained stable from 2001 to 2009 (0.8%), pronounced regional variations have been observed, with infection rates varying from <0.5% to >40% [2]. In Brazil, the prevalence of HIV-1 infection in individuals aged 15–49 years old is estimated to be 0.6%, varying from 0.8% to 0.4% in men and women, respectively [3].

In the first half of the last century, HIV-1 group M branched into genetic subtypes but remained confined to western-central Africa [4]. However, in the second half, the epidemic became global, resulting in a differential global distribution of HIV-1 subtypes and recombinant genomes [5]. The high mutation and viral replication rates associated with reverse transcriptase's error-prone activity result in large genetic variability of HIV strains worldwide. Although the global and regional distributions of individual subtypes and recombinants have been stable under a coarse-grained analysis, Circulating Recombinant Forms (CRFs) play an increasing role in the HIV pandemic. Roughly, 55 CRFs have been described so far (Los Alamos National Laboratory, http://www.hiv.lanl.gov) [5]. Data from Brazil have been available since the nineties showing a predominance of subtype B in distinct regions of the country, followed by subtype F, and an uncountable number of Unique Recombinant Forms involving subtypes B and F (URF_BF), exception made for the Southern states, where subtype C and CRF31_BC predominate [6]. Indeed, HIV-1 diversity remains a major challenge for the development of an HIV vaccine [5].

Despite the advances in antiretroviral therapy (ART) that have revolutionized HIV management and contributed to the observed decline of regional epidemics [7], [8], [9], antiretroviral resistance has emerged in all settings in which such drugs have been prescribed. In fact, transmitted resistance represents a permanent challenge for HIV control due to its impact on treatment efficacy and clinical outcomes. Data from reports around the world have shown high levels of transmitted antiretroviral resistance over the years in high-income countries [10], [11], [12], [13]. A gap remains for most middle and low-income were such data are sorely needed in the context of treatment scale-up.

Brazil is a middle-income country and since 1996 has implemented a policy of universal access to highly active antiretroviral therapy (HAART). Over 300.000 HIV-1 infected individuals are currently under treatment. So far, national cross-sectional surveys pointed out to an intermediate level of HIV-1 primary drug resistance (5-15%) [14], [15]. In this paper, we aimed to assess HIV subtype diversity and transmitted resistance prevalence over time in newly diagnosed individuals, in the Rio de Janeiro metropolitan area.

## Materials and Methods

### Ethics Statement

This study was approved by the Evandro Chagas Clinical Research Institute - IRB (CAAE–0032.0.009.000-04). The individuals were interviewed at the VCT sites and signed the informed consent. Written consent of relatives or guardians were obtained on behalf of adolescents (aged 15–17 years old) included in the study. Personal identifications were systematically removed from data.

### Patient Population

From 2005 to 2007, 27,807 individuals seeking HIV diagnosis in four Voluntary Counseling and testing sites (VCTs) located in the Rio de Janeiro metropolitan region (i.e. the city of Rio de Janeiro and its outskirts) were enrolled in this study. Blood samples were obtained and routine serological assays to assess HIV infection were performed. Seropositive samples from each year were further tested in order to differentiate recent seroconverters (RS) and long-term seroconverters (LTS), using the BED-CEIA protocol [16] according to the manufacturer's instructions. One hundred-forty four consecutive samples of RS (2005–2007) as well as a random subset of 102 LTS samples diagnosed in 2005 [17] were submitted to molecular protocols.

### HIV-1 subtyping

DNA samples were sequenced for a fragment of the polymerase region covering the protease and part of the reverse transcriptase, as previously described [18]. The DNASTAR package was used for sequence edition [19]. HIV-1 subtypes and *Pol* recombinant forms were determined by Rega HIV-1 Subtyping Tool website [22], accessed in November 2012, and phylogenetic inferences using the Neighbor-Joining algorithm under Kimura-2 parameters, nucleotide substitution model [20] through Mega 5.2 software packages [21]. Recombinant profiles were inferred by bootscanning analyses with a sliding window of 350 bp, steps of 10 bp and Kimura-2 parameters model using SimPlot 3.5.1 software [23]. The sequences were submitted to GenBank and the accession numbers are KF921970 to KF922070 for those obtained from the LTS group and KF922071 to KF922214 for the RS group.

### HIV-1 transmitted resistance

HIV-1 transmitted resistance was evaluated according to the Calibrated Population Resistance (CPR) Tool Version 6.0. The CPR tool is a program used to analyze HIV-1 sequences, and constitutes a standard approach to determine the sequences containing a mutation suggestive of transmitted HIV-1 drug resistance, generating a list of standard surveillance drug resistance mutations (SDRMs) [24]. This approach was endorsed by the World Health Organization (WHO) for epidemiological surveillance of transmitted HIVDR [25], [26]. The analysis was done using the CPR website http://cpr.stanford.edu/cpr.cgi accessed in November 2013.

### Data Analysis

Two major groups (LTS of 2005, RS from 2005 to 2007) were evaluated according to VCT location, gender, sexual practice for men, pregnancy in women and age. Contingency tables and respective statistics were used (i.e. chi-square or Fisher's exact test for categorical variables). An alpha level of 0.05 was chosen to define statistical significance.

## Results

**Figure 1:**
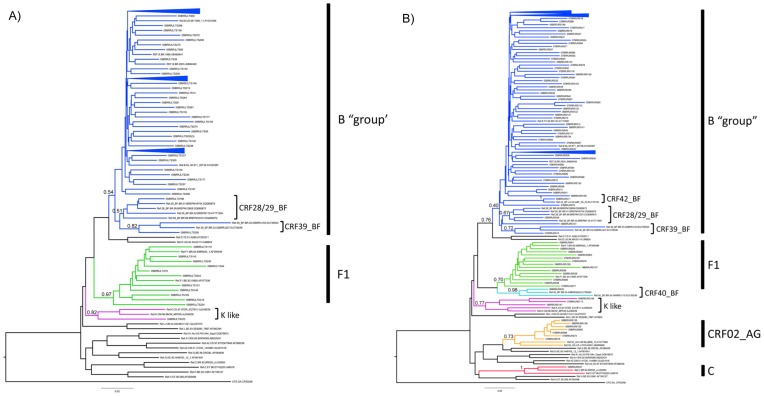
Phylogenetic tree analysis of the HIV-1 pol region, encompassing the protease and part of the reverse transcriptase, obtained from (A) long term seroconverters (LTS) and (B) recent seroconverters (RS) from Rio de Janeiro, Brazil. The phylogenetic inferences were performed by the Neighbor-Joining algorithm under Kimura-2 parameters, nucleotide substitution model using MEGA 5.2 package. Reference sequences from the major HIV-1 subtypes and CRFs BF and CRF02_AG were also included. The scale represents number of substitutions per site. The major subtype and CRF clusters are indicated in different colors. Subtype B samples as well as CRF 28, 29 and 39 were included in the Subtype B group.

HIV-1 Pol genotyping were performed for 102 LTS identified in 2005 and for 144 RS recruited from 2005–2007. Phylogenetic analyses of HIV-1 samples obtained for both groups are presented in figures 1A and 1B, respectively. Overall, subtype B (78.0%) infections prevailed over time, without significant differences (p = 0.12) between the LTS (2005) and RS (2005–2007) subgroups (83.3% *vs* 74.3%). Among non-B subtype samples, most were from subtype F1 (24 in 25 samples) and one RS sample was assigned as subtype C. During the period under analysis, approximately one in every nine samples presented a recombinant profile between different subtypes. Among them, URF_BF samples were detected in both LTS (n = 2) and RS (n = 6) subgroups. Moreover, URF_CD as well as URF_BD (two samples) and URF_CF were identified among the LTS and RS subgroups, respectively. These samples were not included in the phylogenetic analyses to improve the bootstrap values of the B and F branch clusters.

**Figure 2:**
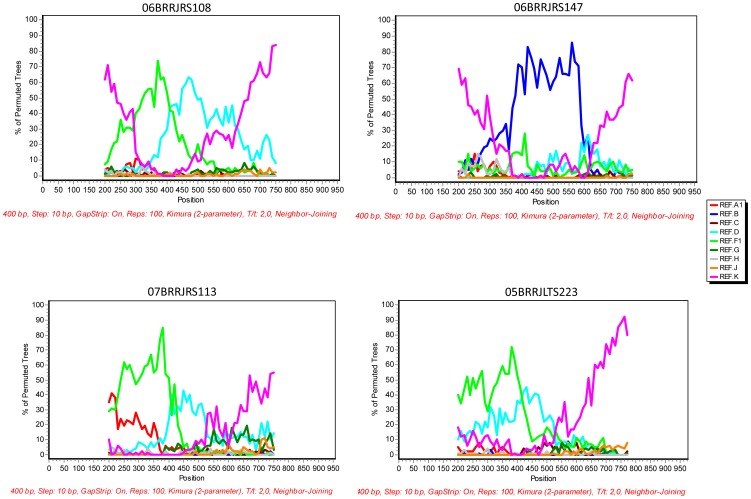
Bootscanning plot of HIV-1 recombinant samples bearing a fragment of subtype K in the polymerase region obtained from LTS (n = 1) and RS (n = 3) individuals. Recombinant profiles were inferred using a sliding window of 350-2 parameters model using SimPlot 3.5.1 software. Referral samples corresponding to the major HIV-1 subtypes, indicated by different colors were included in the analyses.

Unique infections due to CRF39_BF and CRF28/29_BF have been observed in LTS and RS subgroups, whereas CRF40_BF and CRF42_BF were detected in RS HIV-1 positive individuals (figures 1A and 1B). The Pol fragment analyzed in the present study was not informative enough to discriminate between CRF28_BF and CRF29_BF and two HIV-1 samples obtained from LTS and RS individuals were classified as CRF28/29_BF. Moreover, four samples (one from LTS and three from RS groups) clustered in the phylogenetic analyses with high bootstrap values with subtype K references. Their URF_K like recombinant profiles were further defined by bootscanning analyses (Figure 2) and were found to correspond to mosaic genomes encompassing subtypes B, F and K.

It is of note that seven cases of CRF02_AG were observed among the 144 RS samples obtained in the period of 2005–2007, what contrasted with the absence of any of such cases among the LTS (0/102) — indicating an increase in the frequency of this CRF in our region over time (p = 0.04).

A summary of the *pol* genetic profiles of the HIV-1 samples obtained from RS and LTS individuals, as well as their association with gender and sexual risk behaviors are presented in Table 1. In order to cross-compare their prevalence in the two subgroups, samples were grouped as “pure subtypes”, defined as those without recombinant genomes in the region under analysis (such as subtypes B, F1 and C), B and non-B subtypes, and URF and CRF “recombinant genomes” pooled or as two categories. Differences between categories found to be statistically significant are highlighted in bold.

**Table 1:** Diversity in *pol* sequences obtained from HIV-1 seropositive individuals diagnosed in four VCTs located in metropolitan area of Rio de Janeiro, Brazil, from 2005 to 2007.

	2005 Prevalent	2005–2007 Incident	Overall
	N (%)	N (%)	N (%)
OVERALL	102 (41.5)	144 (58.5)	246 (100.0)
**B**	85 (83.3)	107 (74.3)	192 (78.0)
**F1**	11 (10.7)	13 (9.0)	24 (9.8)
**BF1**	4 (4.0)	10 (6.9)	14 (5.7)
**CRF_02AG**	0 (0.0)	7 (4.9)	7 (2.8)
**K recombinants**	1 (1.0)	3 (2.1)	4 (1.6)
***Other forms***	1 (1.0) *	4 (2.8) **	5 (2.1)
***Subtype***			
Pure	**96 (94.1)** #	**121 (84.0)** #	217 (88.2)
Recombinant	**6 (5.9)** #	**23 (16.0)** #	29 (11.8)
***Pure Subtype***			
B	85 (88.5)	107 (88.4)	192 (88.5)
other than B	11 (11.5)	14 (11.6)	25 (11.5)
***Recombinant***			
URF	4 (66.7)	12 (52.2)	16 (55.2)
CRF (related)	2*** (33.3)	11**** (47.8)	13 (44.8)
			
**MALE - FEMALE**	56 (54.9)−46 (45.1)	74 (51.4)−70 (48.6)	130 (52.8)−116 (47.2)
***Subtype***			
Pure	**54 (96.4)** ##−42 (91.3)	**61 (82.4)** ##−60 (85.7)	115 (88.5)−102 (87.9)
Recombinant	**2 (3.6)** ##−4 (18.7)	**13 (17.6)** ##−10 (14.3)	15 (11.5)−14 (12.1)
***Pure Subtype***			
B	48 (88.9)−37 (88.1)	54 (88.5)−53 (88.3)	102 (88.7)−90 (88.2)
other than B	6 (11.1)−5 (11.9)	7 (11.5)−7 (11.7)	13 (11.3)−12 (11.8)
***Recombinant***			
URF	2 (100.0)−2 (50.0)	5 (38.5)−7 (70.0)	7 (46.7)−9 (64.3)
CRF (related)	0 (0.0)−2 (50.0)	8 (61.5)−3 (30.0)	8 (53.3)−5 (35.7)
			
MSM - HETERO ###	19 (33.9)−37 (66.1)	32 (50.8)−31 (49.2)	51 (42.8)−68 (57.2)
***Subtype***			
Pure	18 (94.7)−36 (97.3)	26 (81.3)−27 (87.1)	44 (86.3)−63 (92.7)
Recombinant	1 (5.3)−1 (2.7)	6 (18.7)−4 (12.9)	7 (13.7)−5 (7.3)
***Pure Subtype***			
B	17 (94.5)−31 (86.1)	23 (88.5)−23 (85.2)	40 (90.9)−54 (85.7)
other than B	1 (5.5)−5 (13.9)	3 (11.5)−4 (14.8)	4 (9.1)−9 (14.3)
***Recombinant***			
URF	1 (100.0)−1 (100.0)	3 (50.0)−0 (0.0)	4 (57.1)−1 (20.0)
CRF (related)	0 (0.0)−0 (0.0)	3 (50.0)−4 (100.0)	3 (42.9)−4 (80.0)

* URF_CD (1).

** subtype C (1), URF_CF (1), URF_BD (2).

*** CRF28_BF/CRF29_BF (1), CRF39_BF (1).

**** CRF02_AG (7), CRF28_BF/CRF29_BF (1), CRF39_BF (1), CRF40_BF (1), CRF42_BF (1).

# P value = 0.016.

## P value = 0.014.

### 11 male volunteers didn't want to express information about their sexual practices.

Although not statistically significant, the URFs:CRFs ratio varied over time, decreasing from 2∶1 (4 URF and 2 CRF-related samples), in the LTS, to almost 1∶1 in the RS subgroup (12 URF and 11 CRF-related samples), mainly due to an increase of CRF02_AG related samples over time. It is of note, however, that recombinant genomes became more prevalent over time, comparing the LTS and RS groups (p = 0.016), as well as among men compared to women (3.6% *vs* 17.6%; p = 0.013).

The analysis of transmitted drug resistance mutations (TDRM) showed that 15% of samples had at least one major mutation associated with drug resistance (Table 2). Protease inhibitors (PI) TDMRs were found in 4.9% of the sequences. Regarding the reverse transcriptase inhibitors, 8.9% and 7.3% of the sequences showed mutations that conferred resistance to nucleoside analogues (NRTIs) and to non-nucleoside analogues (NNRTIs), respectively. Moreover, 3.7% and 0.8% of the samples had resistance mutations to NRTIs and NNRTIs and to the three antiretroviral (ARV) classes, respectively.

**Table 2:** Frequency of HIV-1 positive individuals carrying SDRM distributed according to drug class, time of infection, gender, sexual practice for men, pregnancy, location, age and subtype in sequences obtained in four VCTs located in metropolitan area of Rio de Janeiro, Brazil.

	N analysed (%)	any	PI	NRTI	NNRTI	NRTI + NNRTI	NRTI + NNRTI + PI
***OVERALL***	246 (100)	15.0	4.9	8.9	7.3	3.7	0.8
***Time of Infection***							
Recent (RS)	144 (58.5)	14.6	3.5	10.4	6.3	3.5	0
Long Term (LTS)	102 (41.5)	15.7	6.9	6.9	8.8	3.9	2.0
***Over Time***							
LTS in 2005	102 (41.5)	15.7	6.9	6.9	8.8	3.9	2.0
RS in 2005	54 (22.0)	16.7	3.7	13.0	7.4	3.7	0
RS in 2006	38 (15.4)	15.8	7.9	7.9	7.9	5.3	0
RS in 2007	52 (21.1)	11.5	0	9.6	3.8	1.9	0
***Gender***							
Male	130 (52.8)	16.2	4.6	9.2	9.2	4.6	0.8
Female	116 (47.2)	13.8	5.2	8.6	5.2	2.6	0.9
***Sexual practice (men)***							
MSM	51 (42.8)	25.5	5.9	11.8	13.7	3.9	0
Heterosexual	68 (57.2)	11.8	4.4	8.8	7.4	5.9	1.5
***Pregnancy (women)***							
Yes	11 (9.5)	0	0	0	0	0	0
No	105 (90.5)	15.2	5.7	9.5	5.7	2.9	1.0
***VCT***							
Madureira	54 (22.0)	16.7	9.3	13.0	9.3	7.4	1.9
Nova Iguaçu	112 (45.5)	11.6	4.5	6.3	6.3	3.6	0.9
Caxias	53 (21.5)	20.7	3.8	7.5	11.3	1.9	0
São Gonçalo	27 (11.0)	14.8	0	14.8	0	0	0
***Age***							
15 to 24 ys.	39 (16.6)	17.9	5.1	7.7	5.1	0	0
25 to 49 ys.	173 (73.6)	14.5	4.6	8.7	7.5	4.0	0.6
More than 49 ys.	23 (9.8)	21.7	8.7	17.4	13.0	9.7	4.3
***Subtype***							
B	192	16.7	6.3	10.4	7.8	4.2	1.0
F1	24	16.7	0.0	8.3	12.5	4.2	0
Recombinants with B	16	12.5	6.3	6.3	0	0	0
AG	7	0	0	0	0	0	0

Comparisons between RS *vs* LTS, in terms of gender, sexual behaviors, pregnancy/non-pregnancy, VCTs location and age did not show any significant difference. When evaluating resistance according to the HIV subtype, the same rate of 16.7% was found for subtypes B and F, whereas 12.5% was observed for those samples presenting recombinant genomes including a fragment of subtype B (2 samples out of fourteen BF and two BD). No TDRM was detected for CRF02_AG samples (seven). These data are summarized in Table 2.

Most resistant mutations found in this study were NRTI mutations. The overall prevalence for this class of mutations was 15.9%, with 12.7% in LTS *vs* 18.1% in RS. The most frequently found NRTI SDMR was M184V/I,, corresponding to almost 30% of the NRTI SDMRs. The second most prevalent mutation in this group was K219R (n = 7; one in LTS and six in RS). The second most prevalent group was SDMR for NNRTI, being 10,8% in the LTS subgroup and 6.3% characterized in the RS group. K103N was the most prevalent NNRTI mutation (n = 10, five in each group), followed by Y181C/L (n = 4) and Y188C (n = 3). The M46L/I (n = 5, three in RS) and L90M (n = 3, two in LTS) were the most frequently observed PI mutations. The frequency of mutations for this drug class is more than two times higher in LTS than in the RS subgroup (9.8% *vs* 4.2%).

## Discussion

In order to assess HIV-1 diversity and TDRM over time, we analyzed samples from recently infected individuals in 2005–2007, as well as samples collected in 2005 and defined as long-term infections from four large VCTs in Rio de Janeiro, Brazil. Our results show that subtype B remains the most prevalent subtype, followed by recombinants — mostly BF1, and subtype F1, as previously described [17].

A higher proportion of recombinant forms (16.0% in RS *vs* 5.9% in LTS) and CRF-related samples (compared to URFs) were observed over time, corroborating recent data published by WHO-UNAIDS [5], documenting a worldwide increase in the proportion of circulating recombinant forms, with a concomitant reduction of the URFs and an increase of overall recombinant genomes.

Interestingly, the observed trend for Rio de Janeiro seems to be secondary to the increase of CRF over time among men, heterosexual (12.9% in RS *vs* 2.7% in LTS) and MSM (18.7% *vs* 5.3%). We hypothesize that men from Rio de Janeiro Metropolitan area (a major touristic destination and an important commercial hub) may constitute a population with a high level of imported infections, which may modulate the local epidemic dynamics, with a higher viral diversity as one of its consequences. This hypothesis seems to be corroborated by the relevant proportion (30% or 7/23) of new infections with recombinants that were classified as related to CRF_02AG. These infections were more prevalent among men (5/7) and correspond to an increasing proportion of recombinant HIV-1 samples among RS over the years (20.1% in 2005 and 33.3% in 2006 and 2007).

CRF_02AG is the fourth most prevalent recombinant form around the world, with a substantial diffusion in West Africa and, to a lesser extent, Central Africa, North Africa, and the Middle East [5]. So far there have been few reports in Brazil [27], [28], and this is consistent with our findings, as all CRF_02AG samples were found among individuals defined as RS. Indeed, in a previous study from our group, six CRF02_AG samples were identified in Rio de Janeiro from 2004 to 2011, evidencing multiple introductions and progressive dissemination across local transmission networks, among patients referred to HIV genotyping by their clinicians [29]. Another study also documented the introduction of CRF02_AG in the Amazonian region, northern Brazil [30]. The occurrence of four recombinants that had a fragment of subtype K corroborates as well our hypothesis. Three of them were obtained from HIV-positive individuals characterized as RS, and one from LTS, previously described [17]. To date, only three reports of isolated cases of subtype K-related genomes have been published among HIV-1 samples from Brazil [17], [31], [32].

HIV-1 subtype K was originally described in samples from Cameroon and the Democratic Republic of Congo [33] and, since then, several CRFs containing subtype K fragments along the genome have been described [http://www.hiv.lanl.gov/content/sequence/HIV/CRFs/CRFs.html]. Among them CRF04_cpx, CRF06_cpx, CRF45_cpx and CRF49_cpx have a subtype K fragment in the polymerase region, as our samples. The relationships between our samples and these described CRFs should be better understood by additional studies.

Our data suggest that the overall levels of transmitted drug resistance remained stable but high (ranging from 11.5% to 16.7%) during the entire study period. In the beginning of the HAART era (1996–8), two studies conducted in Brazil [34], [35] showed an overall prevalence rate for transmitted resistance that ranged from 0.0% to 0.9%; resistance was mostly to NRTIs [34]. In 2001, a large study assessing data from eight different Brazilian states found an overall prevalence of 6.6%, with an uniform distribution among different drug classes (2.4% for NRTIs, 2.0% for NNRTIs and 2.2% for PIs) [14]. More recently, surveys have reported intermediate to high levels of primary resistance to at least one class of antiretroviral drugs, varying from 7.0% to 21.4% [15], [30], [31], [36]–[38].

Our results are in agreement with recent reports that documented an increasing transmitted resistance level in Brazil. Taken together, these data may contribute to the ongoing debate about the need of implementing resistance testing before starting ART, at least in settings within the country where high rates of transmitted resistance have been described. One must observe, however, that in a continental size country, with a pronounced social and geographic diversity, high levels of resistance may reflect the availability of molecular surveillance studies in some specific settings rather than actual worst-case scenarios. Most studies carried out so far in Brazil have focused on areas where a better logistical and academic infrastructure is available, what may or may not be coincident with areas where resistance might be actually higher and of special concern.

No difference between men and women was made evident, but the prevalence of transmitted resistance among MSM was higher than among heterosexual men (25.5% *vs* 11.8%). This high level of transmitted resistance aligns with the findings from a recent respondent-driven sampling study of MSM from nine Brazilian cities, in which a 21.4% rate was found [38]. This can be due to the longer time of ARV exposure among MSM, as this population has been the most affected in our country since the beginning of the HIV epidemic. In consequence, a high proportion of MSM are likely to be exposed to multiple ARVs for a longer period, increasing the chances of new infections harboring a resistant virus in this population.

Among samples containing CRF_02AG, the absence of resistance could be explained by their probable origin. They could be imported from the African continent, from where Brazil has received many immigrants in recent years. In most regions of Africa, ARV treatment remains limited and in need for further scaling-up.

The prevalence of resistance was found to be similar among B and F subtype infections, despite some variation by specific drug classes.

One must be cautious respecting the generazibility of such findings, in the context of a huge and heterogeneous country. Additional molecular surveillance studies must be carried out in other regions of the country and are sorely needed for specific areas, such as southern Brazil, where the most prevalent subtypes markedly differ from those observed in Rio de Janeiro and other regions [5], [6], [14], [39]–[42].

Although the study was conducted following all recommendations on surveillance studies issued by the CDC [43], one of its potential limitations is related to the possibility of detecting “false-recent infections”, misclassified by BED-CEIA in contexts of high viral diversity.

Our findings reinforce the concept that has gained momentum worldwide that HIV-1 viral diversity and transmitted resistance have become increasingly complex. It is therefore highly recommended that, at least in some localities/regions of the country, primary resistance should be performed to better inform treatment decisions.

## References

[1] UNAIDS (2012) Global report: UNAIDS report on the global AIDS epidemic 2012.

[2] UNAIDS (2010) Global report: UNAIDS report on the global AIDS epidemic 2010.

[3] Szwarcwald CL, Barbosa Junior A, Souza-Junior PR, Lemos KR, Frias PG, et al (2008) HIV testing during pregnancy: use of secondary data to estimate 2006 test coverage and prevalence in Brazil. Braz J Infect Dis 12: 167–172. 18833398 10.1590/s1413-86702008000300002

[4] Worobey M, Gemmel M, Teuwen DE, Haselkorn T, Kunstman K, et al (2008) Direct evidence of extensive diversity of HIV-1 in Kinshasa by 1960. Nature 455: 661–664. 18833279 10.1038/nature07390 PMC3682493

[5] Hemelaar J, Gouws E, Ghys PD, Osmanov S (2011) Global trends in molecular epidemiology of HIV-1 during 2000–2007. Aids 25: 679–689. 21297424 10.1097/QAD.0b013e328342ff93 PMC3755761

[6] Almeida SE, de Medeiros RM, Junqueira DM, Graf T, Passaes CP, et al (2012) Temporal dynamics of HIV-1 circulating subtypes in distinct exposure categories in southern brazil. Virol J 9: 306. 23234345 10.1186/1743-422X-9-306 PMC3547702

[7] Palella FJ Jr, Delaney KM, Moorman AC, Loveless MO, Fuhrer J, et al (1998) Declining morbidity and mortality among patients with advanced human immunodeficiency virus infection. HIV Outpatient Study Investigators. N Engl J Med 338: 853–860. 9516219 10.1056/NEJM199803263381301

[8] Montaner JS, Lima VD, Barrios R, Yip B, Wood E, et al (2010) Association of highly active antiretroviral therapy coverage, population viral load, and yearly new HIV diagnoses in British Columbia, Canada: a population-based study. Lancet 376: 532–539. 20638713 10.1016/S0140-6736(10)60936-1 PMC2996043

[9] Johnston KM, Levy AR, Lima VD, Hogg RS, Tyndall MW, et al (2010) Expanding access to HAART: a cost-effective approach for treating and preventing HIV. Aids 24: 1929–1935. 20588171 10.1097/QAD.0b013e32833af85d

[10] Tamalet C, Fantini J, Tourres C, Yahi N (2003) Resistance of HIV-1 to multiple antiretroviral drugs in France: a 6-year survey (1997–2002) based on an analysis of over 7000 genotypes. Aids 17: 2383–2388. 14571191 10.1097/01.aids.0000076341.42412.59

[11] Scott P, Arnold E, Evans B, Pozniak A, Moyle G, et al (2004) Surveillance of HIV antiretroviral drug resistance in treated individuals in England: 1998–2000. J Antimicrob Chemother 53: 469–473. 14749345 10.1093/jac/dkh102

[12] Richman DD, Morton SC, Wrin T, Hellmann N, Berry S, et al (2004) The prevalence of antiretroviral drug resistance in the United States. Aids 18: 1393–1401. 15199315 10.1097/01.aids.0000131310.52526.c7

[13] Taniguchi T, Nurutdinova D, Grubb JR, Önen NF, Shacham E, et al (2012) Transmitted drug-resistant HIV type 1 remains prevalent and impacts virologic outcomes despite genotype-guided antiretroviral therapy. AIDS Res Hum Retroviruses 28: 259–64. 21877906 10.1089/aid.2011.0022

[14] Brindeiro RM, Diaz RS, Sabino EC, Morgado MG, Pires IL, et al (2003) Brazilian Network for HIV Drug Resistance Surveillance (HIV-BResNet): a survey of chronically infected individuals. Aids 17: 1063–1069. 12700457 10.1097/00002030-200305020-00016

[15] Inocencio LA, Pereira AA, Sucupira MC, Fernandez JC, Jorge CP, et al (2009) Brazilian Network for HIV Drug Resistance Surveillance: a survey of individuals recently diagnosed with HIV. J Int AIDS Soc 12: 20. 19765271 10.1186/1758-2652-12-20 PMC2759910

[16] Dobbs T, Kennedy S, Pau CP, McDougal JS, Parekh BS (2004) Performance characteristics of the immunoglobulin G-capture BED-enzyme immunoassay, an assay to detect recent human immunodeficiency virus type 1 seroconversion. J Clin Microbiol 42: 2623–2628. 15184443 10.1128/JCM.42.6.2623-2628.2004 PMC427871

[17] de Castro CA, Grinsztejn B, Veloso VG, Bastos FI, Pilotto JH, et al (2010) Prevalence, estimated HIV-1 incidence and viral diversity among people seeking voluntary counseling and testing services in Rio de Janeiro, Brazil. BMC Infect Dis 10: 224. 20667113 10.1186/1471-2334-10-224 PMC2919548

[18] Eyer-Silva WA, Morgado MG (2006) Molecular epidemiology of HIV-1 infection in a small Brazilian county: usefulness of envelope and polymerase sequences to epidemiologic studies. J Acquir Immune Defic Syndr 41: 664–670. 16652042 10.1097/01.qai.0000194736.66322.02

[19] Burland TG (2000) DNASTAR's Lasergene sequence analysis software. Methods Mol Biol 132: 71–91. 10547832 10.1385/1-59259-192-2:71

[20] Saitou N, Nei M (1987) The neighbor-joining method: a new method for reconstructing phylogenetic trees. Mol Biol Evol 4: 406–425. 3447015 10.1093/oxfordjournals.molbev.a040454

[21] Kumar S, Tamura K, Nei M (2004) MEGA3: Integrated software for Molecular Evolutionary Genetics Analysis and sequence alignment. Brief Bioinform 5: 150–163. 15260895 10.1093/bib/5.2.150

[22] de Oliveira T, Deforche K, Cassol S, Salminen M, Paraskevis D, et al (2005) An automated genotyping system for analysis of HIV-1 and other microbial sequences. Bioinformatics 21: 3797–3800. 16076886 10.1093/bioinformatics/bti607

[23] Lole KS, Bollinger RC, Paranjape RS, Gadkari D, Kulkari D, et al (1999) Full-length human immunodeficiency virus type 1 genomes from subtype C-infected seroconverters in India, with evidence of intersubtype recombination. J Virol 73(1): 152–160. 9847317 10.1128/jvi.73.1.152-160.1999 PMC103818

[24] Gifford RJ, Liu TF, Rhee SY, Kiuchi M, Hue S, et al (2009) The calibrated population resistance tool: standardized genotypic estimation of transmitted HIV-1 drug resistance. Bioinformatics 25: 1197–1198. 19304876 10.1093/bioinformatics/btp134 PMC2672634

[25] Shafer RW, Rhee SY, Bennett DE (2008) Consensus drug resistance mutations for epidemiological surveillance: basic principles and potential controversies. Antivir Ther 13 Suppl 2 59–68. 18575192 PMC4388302

[26] Bennett DE, Myatt M, Bertagnolio S, Sutherland D, Gilks CF (2008) Recommendations for surveillance of transmitted HIV drug resistance in countries scaling up antiretroviral treatment. Antivir Ther 13 Suppl 2 25–36. 18575189

[27] Pires IL, Soares MA, Speranza FA, Ishii SK, Vieira MC, et al (2004) Prevalence of human immunodeficiency virus drug resistance mutations and subtypes in drug-naive, infected individuals in the army health service of Rio de Janeiro, Brazil. J Clin Microbiol 42: 426–430.26. 14715797 10.1128/JCM.42.1.426-430.2004 PMC321664

[28] Barreto CC, Nishyia A, Araujo LV, Ferreira JE, Busch MP, et al (2006) Trends in antiretroviral drug resistance and clade distributions among HIV-1–infected blood donors in Sao Paulo, Brazil. J Acquir Immune Defic Syndr 41: 338–341. 16540943 10.1097/01.qai.0000199097.88344.50

[29] Delatorre EO, Bello G, Eyer-Silva WA, Chequer-Fernandez SL, Morgado MG, et al (2012) Evidence of multiple introductions and autochthonous transmission of the HIV type 1 CRF02_AG clade in Brazil. AIDS Res Hum Retroviruses 28: 1369–1372. 22333001 10.1089/AID.2011.0381

[30] Machado LF, Ishak MO, Vallinoto AC, Lemos JA, Azevedo VN, et al (2009) Molecular epidemiology of HIV type 1 in northern Brazil: identification of subtypes C and D and the introduction of CRF02_AG in the Amazon region of Brazil. AIDS Res Hum Retroviruses 25: 961–966. 19795985 10.1089/aid.2009.0027

[31] Pilotto JH, Grinsztejn B, Veloso VG, Velasque LS, Friedman RK, et al (2013) Moderate prevalence of transmitted drug resistance mutations among antiretroviral-naive HIV-infected pregnant women in Rio de Janeiro, Brazil. AIDS Res Hum Retroviruses 29(4): 681–6. 23259924 10.1089/AID.2011.0333

[32] Ferreira FG, Pinto JA, Kakehasi FM, Cleto S, Tupinambás U, et al (2010) Prevalence of primary drug resistance-associated mutations among HIV type 1 vertically Infected children in Belo Horizonte, Brazil. AIDS Res Hum Retroviruses 26(2): 229–32. 20156105 10.1089/aid.2009.0146

[33] Triques K, Bourgeois A, Vidal N, Mpoudi-Ngole E, Mulanga-Kabeya C, et al (2000) Near-full-length genome sequencing of divergent African HIV type 1 subtype F viruses leads to the identification of a new HIV type 1 subtype designated K. AIDS Res Hum Retroviruses 16(2): 139–51. 10659053 10.1089/088922200309485

[34] Brindeiro R, Vanderborght B, Caride E, Correa L, Oravec RM, et al (1999) Sequence diversity of the reverse transcriptase of human immunodeficiency virus type 1 from untreated Brazilian individuals. Antimicrob Agents Chemother 43: 1674–1680. 10390221 10.1128/aac.43.7.1674 PMC89342

[35] Dumans AT, Soares MA, Pieniazek D, Kalish ML, De Vroey V, et al (2002) Prevalence of protease and reverse transcriptase drug resistance mutations over time in drug-naive human immunodeficiency virus type 1-positive individuals in Rio de Janeiro, Brazil. Antimicrob Agents Chemother 46: 3075–3079. 12183276 10.1128/AAC.46.9.3075-3079.2002 PMC127402

[36] Sprinz E, Netto EM, Patelli M, Lima JS, Furtado JJ, et al (2009) Primary antiretroviral drug resistance among HIV type 1-infected individuals in Brazil. AIDS Res Hum Retroviruses 25: 861–867. 19689190 10.1089/aid.2009.0012

[37] Arruda E, Simoes L, Sucupira C, Medeiros M, Arruda E, et al (2011) Short communication: intermediate prevalence of HIV type 1 primary antiretroviral resistance in Ceara State, Northeast Brazil. AIDS Res Hum Retroviruses 27: 153–156. 20929346 10.1089/aid.2010.0028

[38] Bermudez-Aza EH, Kerr LR, Kendall C, Pinho AA, de Mello MB, et al (2011) Antiretroviral drug resistance in a respondent-driven sample of HIV-infected men who have sex with men in Brazil. J Acquir Immune Defic Syndr 57 Suppl 3 S186–192. 21857316 10.1097/QAI.0b013e31821e9c36

[39] Vicente AC, Otsuki K, Silva NB, Castilho MC, Barros FS, et al (2000) The HIV epidemic in the Amazon Basin is driven by prototypic and recombinant HIV-1 subtypes B and F. J Acquir Immune Defic Syndr 23: 327–331. 10836755 10.1097/00126334-200004010-00008

[40] Guimarães ML, Couto-Fernandez JC, Eyer-Silva WA, Teixeira SL, Chequer-Fernandez SL, et al (2010) Analysis of HIV-1 BF pr/rt recombinant strains from Rio de Janeiro/Brazil reveals multiple unrelated mosaic structures. Infect Genet Evol 10(7): 1094–100. 20621204 10.1016/j.meegid.2010.07.001

[41] Gräf T, Passaes CP, Ferreira LG, Grisard EC, Morgado MG, et al (2011) HIV-1 genetic diversity and drug resistance among treatment naïve patients from Southern Brazil: an association of HIV-1 subtypes with exposure categories. J Clin Virol 51(3): 186–91. 21622023 10.1016/j.jcv.2011.04.011

[42] Ferreira AS, Cardoso LP, Stefani MM (2011) Moderate prevalence of transmitted drug resistance and high HIV-1 genetic diversity in patients from Mato Grosso State, Central Western Brazil. J Med Virol 83(8): 1301–7. 21678433 10.1002/jmv.22128

[43] Dobbs T, Parekh BS (2003) Detecting recent human immunodeficiency virus type 1 infection: why and how? MLO Med Lab Obs 35: : 12–14, 16, 19–20 passim; quiz 24–15. 12774433

